# Supplementary material for: Visualizing RNA conformational and architectural heterogeneity in solution
Source: Nat Commun. 2023 Feb 9;14:714. doi: 10.1038/s41467-023-36184-x (PMC9911696; doi:10.1038/s41467-023-36184-x)
Supplement: Supplementary file 1 — Supplementary Information [file 41467_2023_36184_MOESM1_ESM.pdf]

# SUPPLEMENTARY INFORMATION

**Visualizing RNA conformational and architectural heterogeneity in solution**

Jienyu Ding[1], Yun-Tzai Lee[1], Yuba Bhandari[1], Charles D. Schwieters[2], Lixin Fan[3], Ping Yu[1], Sergey G. Tarosov[4], Jason R. Stagno[1], Buyong Ma[5], Ruth Nussinov[5], Alan Rein[6], Jinwei Zhang[7], Yun-Xing Wang[1]

[1]Protein-Nucleic Acid Interaction Section, Center for Structural Biology, National Cancer Institute, Frederick, MD 21702, USA; [2]Computational Biomolecular Magnetic Resonance Core, National Institute of Diabetes and Digestive and Kidney Diseases, National Institutes of Health, Bethesda, MD 20892, USA; [3]Basic Science Program, Frederick National Laboratory for Cancer Research, Small Angle X-ray Scattering Core Facility of National Cancer Institute, National Institutes of Health, Frederick, MD 21702, USA; [4]Biophysical Resource, Center for Structural Biology National Cancer Institute, Frederick, MD 21702, USA; [5]Computational Structural Biology Section, Frederick National Laboratory for Cancer Research in the Laboratory of Cancer Immunometabolism, National Cancer Institute, Frederick, MD 21702, USA; [6]Retrovirus Assembly Section, HIV Dynamics and Replication Program, National Cancer Institute, Frederick, MD 21702, USA; [7]Laboratory of Molecular Biology, National Institute of Diabetes and Digestive and Kidney Diseases, Bethesda, MD 20892, USA.

Corresponding author: Yun-Xing Wang, wangyunx@mail.nih.gov

# Supplementary Discussion

## Atypical Cbl sample prepration

The aCbl RNA samples were generated by *in vitro* T7 RNA polymerase co-transcription (in presence of 0.5 mM AdoCbl ligand) using synthesized linear DNA templeates (IDT, Coralville, Iowa). The trascription products were purified by 10% TBE gel. The target RNA band was eluted from the gel at 4 °C overnight using elution buffer containing 50 mM potassium acetate, pH 7.0, 200 mM KCl, followed by buffer exchange to Tris buffer (25 mM Tris-HCl, pH 8.0, 50 mM KCl, 10 mM $MgCl_2$).

## Immbolization

In all AFM experiments, we used APS (1-(3-aminopropyl)silatrane) to immoblize RNA samples on the mica surface. APS is a modified version of APTES (3-aminopropyltriethoxysilane) with several chemical advantages, such as greater resistance to hydrolysis, broader tolerance to buffers and pH (up to 10), smoother and more uniform surface modification, and a lower surface-charge density[1]. APS-mica is widely used for the non-denaturing immobilization of nucleic acids primarily through the weakly associated electrostatic interactions between protonated amino groups of the APS-mica substrate and the negatively charged nucleic acid backbone. The low surface-charge density of APS-mica allows reliable imaging of nucleic acids, protein-nucleic acid complexes, and other biological samples, which has been demonstrated in many studies[1-3]. Compared to molecules that are labeled, covalently tethered, crystallized under extreme buffer conditions, etc., the solution AFM imaging can be performed under near-native conditions.

## The effects of salt concentration on RNA conformational heterogeneity and activity

The high-resolution AFM images were obtained under low salt buffer (LSB) conditions (10 mM KCl, 1 mM $MgCl_2$). We found, however, that when rCbl was prepared in LSB after gel elution, and stored under these conditions until AFM imaging, the majority of RNA molecules exhibited *Y* conformations and showed very little differences in the absence or presence of ligand (**Supplementary Fig. 1a-b**). We suspect that,

although LSB conditions are optimal for imaging, they are not suitable for maintaining rCbl folding stability over several hours or days. For this reason, rCbl samples were kept in high salt buffer (HSB) conditions (100 mM KCl, 10 mM $MgCl_2$) until just before AFM imaging, at which point they were diluted in LSB and used immediately.

## The effects of RNA and ligand concentrations on conformational heterogeneity

Given the number of various monomer and dimer conformations observed by AFM, the convolution of heat exchange upon ligand binding is likely of greater complexity than can be explained from ITC data, particularly with regard to the effects of RNA and ligand concentrations used in each method, and conformational heterogeneity under equilibrium conditions prior to ligand binding. For instance, at much lower (nM) RNA concentration, as indicated by AFM imaging, conformational changes and dimer formation comprise the bulk of the observed differences induced by ligand binding. Whereas, at the much higher (μM) ITC concentration, the majority of the RNA adopts a bound-like state prior to ligand binding. Therefore, the extent of induced-fit versus conformational selection mode of binding, as it relates to the binding-competent population, ligand-driven conformational changes, and dimer formation, may vary with RNA and ligand concentrations. As such, ITC-, SAXS-, and AFM-derived conformer populations, which are recorded at different RNA and ligand concentrations, are not identical, even though they exhibit consistent trends.

## The singular value decomposition (SVD) Matlab script for ITC data analysis:

Brief description: The Matlab script consists of four parts:

1. ITC data loading: Prepare a matrix A text file for the SVD input.

   Matrix A is a m-by-n matrix. m and n are integer number that represents how many data points constitute ITC thermogram profile and total titration points, respectively.

   For example, 18 ITC titration points will end up with 18 peaks (18 columns).

If each peak is constituted by 60 data points. The input file will be a 60-by-18 matrix, which is the matrix A.

Remove the titration outliers, if any, from matrix A (optional):

A(:, the number of column to be removed) = [];

*Note: the first titration point is usually useless and is removed from the first column of the matrix by A(:,1) = [];

In the “load data section”, you make **point** 18 when you have a total of 18 titration points.

The x is the ligand-to-RNA ratio. The line space will be defined evenly by

x = linespace(lower bound, upper bound, **point**)

then,

Figure (1) will be plotted out to show the superimposition of all individual thermograms.

2. SVD analysis of the matrix A:

svd(A) returns the singular values of the matrix A in descending order and can be decomposed to a left singular vector (U), diagonal (S), and right singular (V) matrices.

[U,S,V] = svd (A)

The singular values of the matrix A can be extracted from the diagonal matrix S.

St = diag(S)

The SVD reconstructed matrix is A1.

The number of singular values “r” utilized to reconstruct A1, is defined by:

r = 3; Assume there are 3 singular value components.

*Note: the number of singular values utilized needs to be specified at the beginning. 3 is a good starting number and can be changed later based on the autocorrelation result.

Ur and Vr are eigenvectors calculated considering the top singular value r.

Ur = U(:,[1:r]);

Sr = S([1:r],[1:r]);

Vr = V(:,[1:r]);

A1=Ur*Sr*Vr'

,where A1 is the reconstructed matrix using significant singular value components.

Calculate fractional percentage of each singular value and plot in Figure (2) shown by a bar chart.

Spercentage = St/Ssum;

Figure (2)

bar(Spercentage,'r','stacked')

3. Autocorrelation analysis:

To judge the number of significant components, the normalized correlation coefficients of individual singular values are calculated and a minimum threshold of 0.75 is used as a selection filter.

The normalized correlation coefficients (Uc and Vc) associated with eigenvectors and each corresponding singular value are calculated by a MATLAB loop function:

**eigenvector U**

```
Uc = zeros(r,1);
for i=1:r
    for ii=1:63
      Uc(i,1)=Uc(i,1)+U(ii,i)*U(ii+1,i);
    end
end
Uc
```

**eigenvector V**

```
Vc = zeros(r,1);
for i=1:r
    for ii=1:point-1
      Vc(i,1)=Vc(i,1)+V(ii,i)*V(ii+1,i);
    end
```

end

Vc

4. Final data outputs:

The eigenvalue projections of the Ur and Vr eigenvectors corresponding the significant singular value components are plotted in Figure (3) and Figure (4), respectively, by

Figure (3)

plot (Ur)

Figure (4)

plot (x,Vr)

Figure (4) can be directly used to proportionally normalize, factorization, and represent the deconvoluted isotherms (as in the main text **Fig. 2f**).

The final reconstructed matrix A1 is plotted out in Figure (5)

Figure (5)

plot (A1)

The overall MATLAB script has been tested by MATLAB and Statistics Toolbox Release 2021a and streamlined in the last page. The output Figures are redrawn and shown in **Supplementary Fig. 11** using GraphPad Prism 8:

```
% ===== Load data ======
filename = 'matrix_file.txt';
point = 18;
 A = importdata(filename);
%A(:,1) = [];
x = linspace(0.0,60.0,point);
Figure (1)
plot (A)
```

```
% ===== SVD analysis =====
[U,S,V] = svd(A);
St = diag(S);
Ssum = sum(St);
Spercentage = St/Ssum;
Figure (2)
bar(Spercentage,'r','stacked')

r = 3;
Ur = U(:,[1:r]);
Sr = S([1:r],[1:r]);
Vr = V(:,[1:r]);

% ===== Autocorrelation =====
% % ===== eigenvector U ======
Uc = zeros(r,1);
for i=1:r
    for ii=1:63
        Uc(i,1)=Uc(i,1)+U(ii,i)*U(ii+1,i);
    end
end
Uc

% % ===== eigenvector V ======
Vc = zeros(r,1);
```

```
for i=1:r
    for ii=1:point-1
        Vc(i,1)=Vc(i,1)+V(ii,i)*V(ii+1,i);
    end
end
Vc
```

**% ===== Output plots =====**

```
A1=Ur*Sr*Vr';
A=U*S*V';

Figure (3)
plot (Ur)
Figure (4)
plot (x,Vr)
Figure (5)
plot (A1)
```

# Supplementary Figures

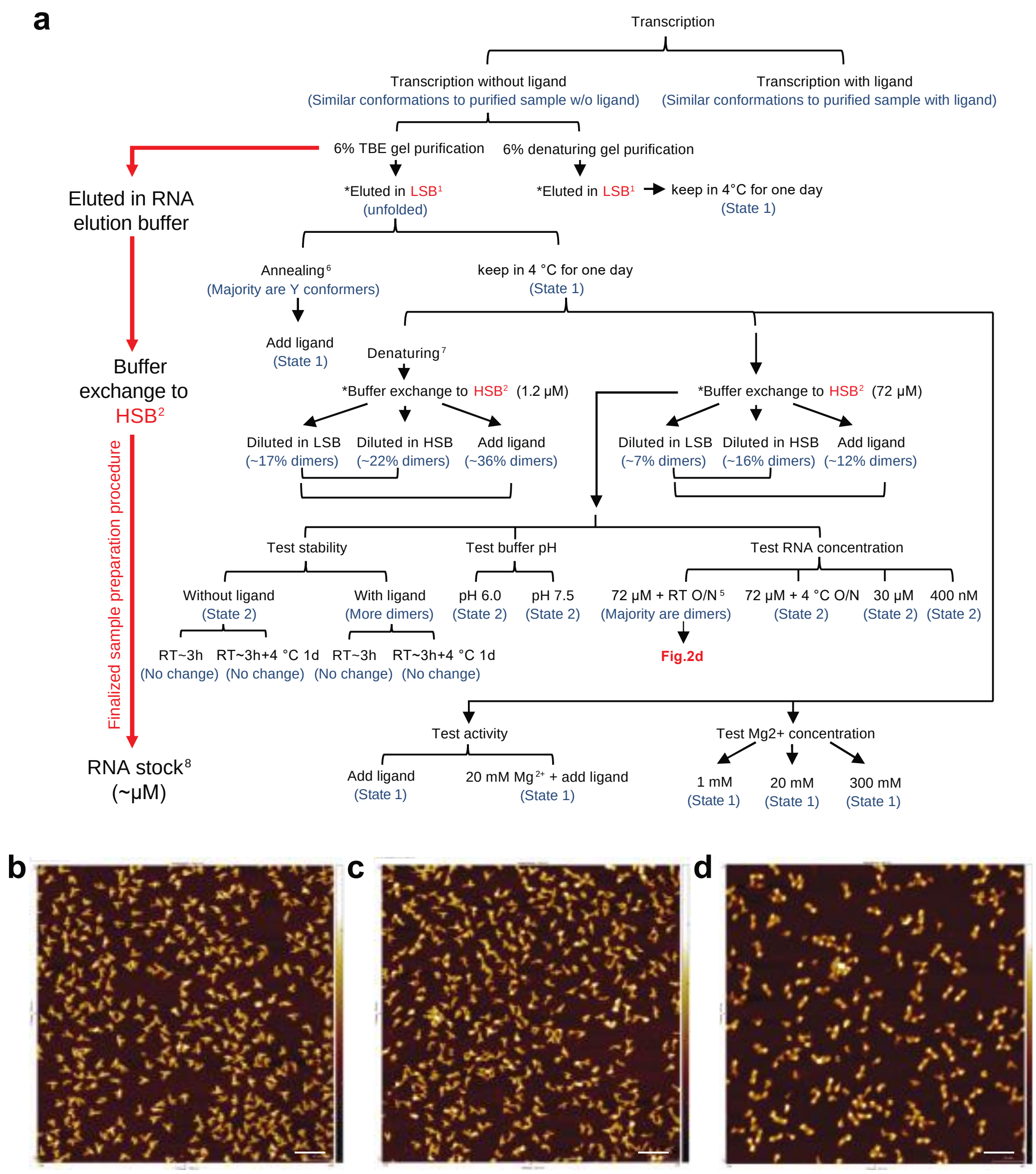


**Supplementary Figure 1 | Extensive study on factors affecting the conformations of rCbl RNA.** (**a**) Illustration of sample preparation protocols with corresponding notes.
[1]LSB: 50 mM MES, pH 6.0, 10 mM KCl, 1 mM $MgCl_2$.
[2]HSB: 10 mM HEPES, pH 7.5, 100 mM KCl, 0.1 mM EDTA, 10 mM $MgCl_2$.
[3]Conformations in purified sample without ligand: mixture of *Y*, *candy*, and *P* monomers (~98%), and various dimers (~2%) (see **Fig. 1a** in main text).

[4]Conformations in purified sample with ligand: mixture of *Y*, *candy*, and *P* monomers (~37%), and various dimers (~63%) (see **Fig. 1b** in main text).
[5]Incubating 72 μM RNA sample under high salt buffer at room temperature overnight results in much more homogeneous conformations (see panel **d**).
[6]Annealing: sample heated at 85 °C for 2 min, followed by snap-cooling on ice.
[7]Denaturing: sample mixed with 8 M urea solution (DEPC H2O + 1 mM EDTA), and heated at 65 °C for 5 min.
[8]Finalized sample preparation procedure for rCbl. All samples presented in main text were prepared in this way. Elution and buffer exchange steps marked with * result in concentration of RNA. (**b-d**) The AFM images for State 1 (**b**), State 2 (**c**), and homogeneous dimers (**d**). The scale bars shown in the right bottom corner of (**b-d**) are 100 nm. The total images recorded for (**a**), (**b**), and (**c**) are 2, 2, 4, respectively.

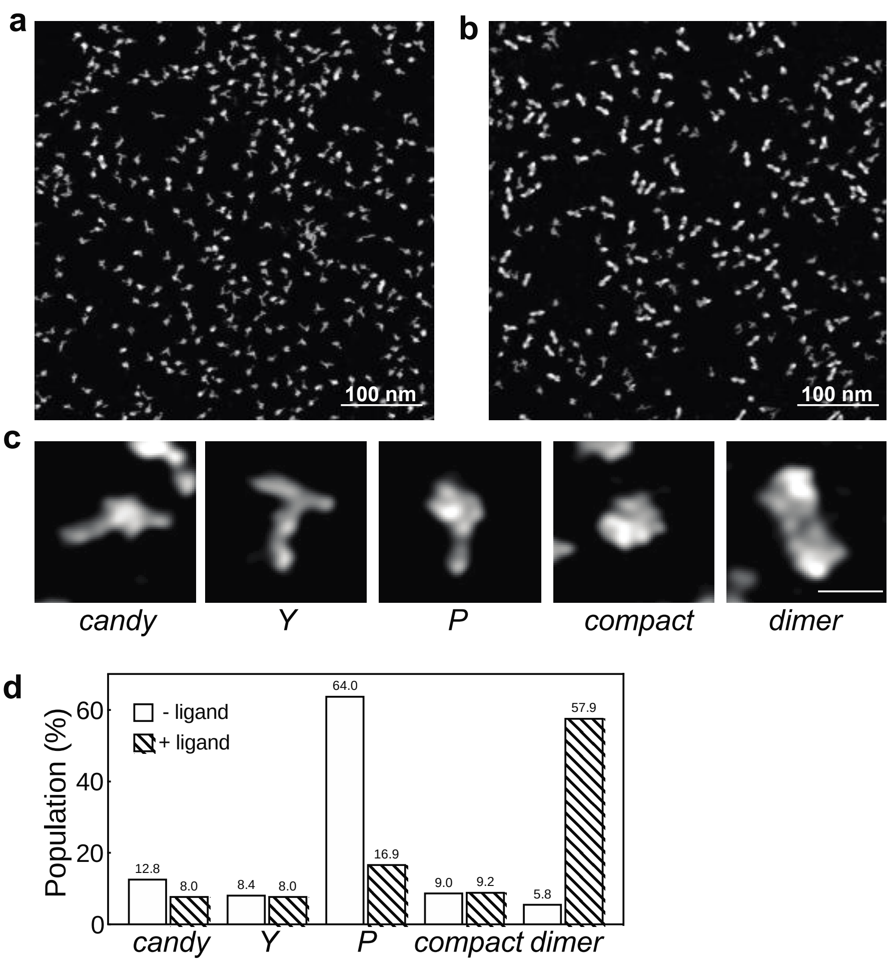


**Supplementary Figure 2 | Conformational heterogeneity of atypical cobalamin riboswitch (aCbl) in solution.** High-resolution AFM images of aCbl in absence (**a**) or presence (**b**) of ligand. A total of 5 images were recorded for aCbl in the absence of ligand, and 4 images for aCbl in the presence of ligand. (**c**) Individual particles cropped from the experimental AFM images shown in (**a-b**), representative of different aCbl conformations (*candy*, *Y*, *P*, *compact*, and *dimer*), which are similar to those observed for rCbl. The scale bar shown on the right bottom corner is 10 nm. (**d**) Tallies of individual conformations in absence (344 particles) or presence (349 particles) of ligand.

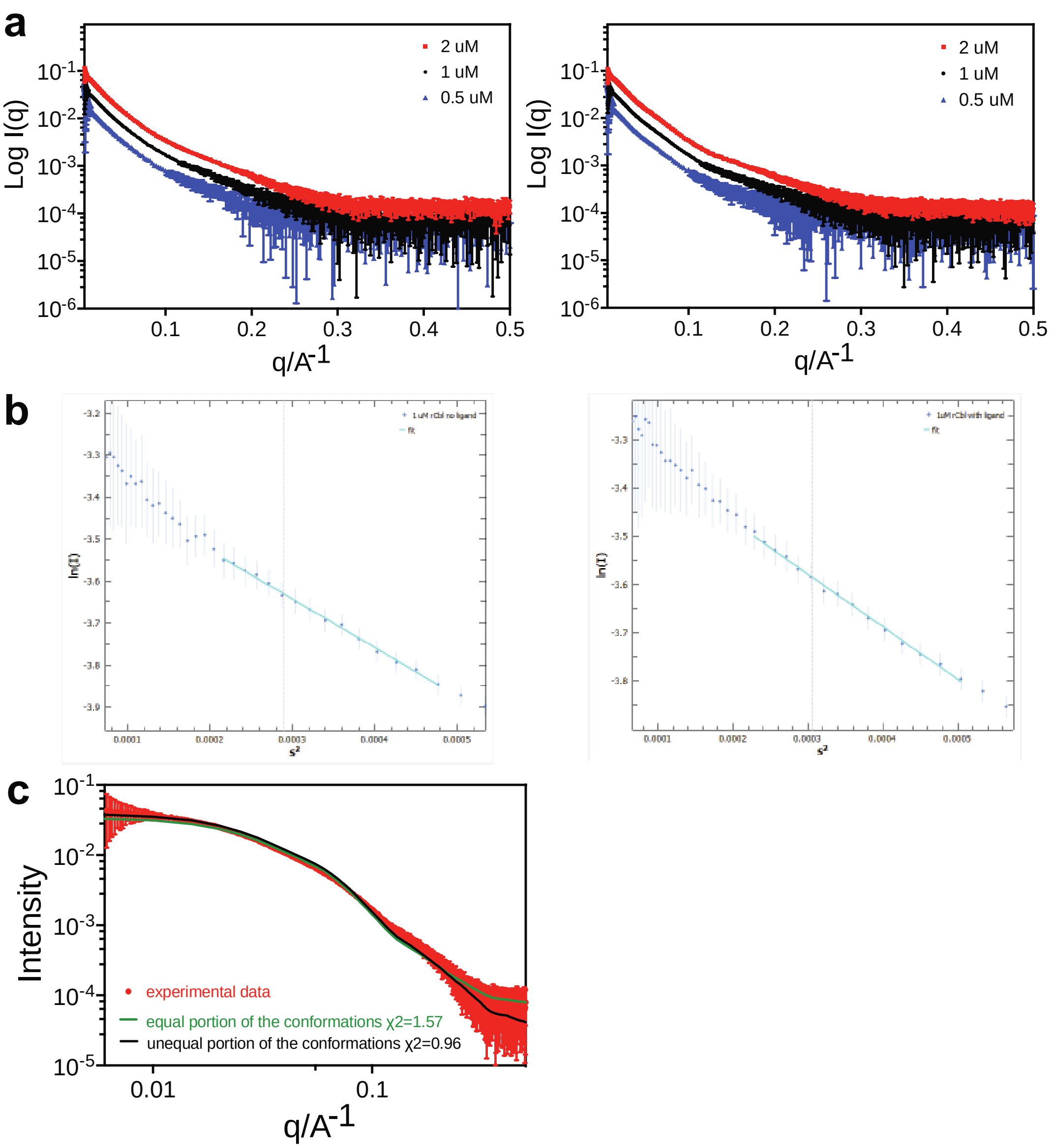


**Supplementary Figure 3 | SAXS results for rCbl in absence or presence of ligand.** (**a-b**) 1D scattering curves of rCbl at different RNA concentrations (**a**) and Guinier plots at 1 µM rCbl (**b**) in absence (left) or presence (right) of ligand. (**c**) Comparison of synthesized SAXS curve using equal populations (green) or weighted populations (black) of all conformers with that of the experimental data (red). The SAXS data was recorded at 1 uM RNA concentration in the presence of ligand. Data are presented as mean value +/- error (propagation of uncertainty) as bar and whisker (n=223 measurements). Source data are provided as a Source Data file.

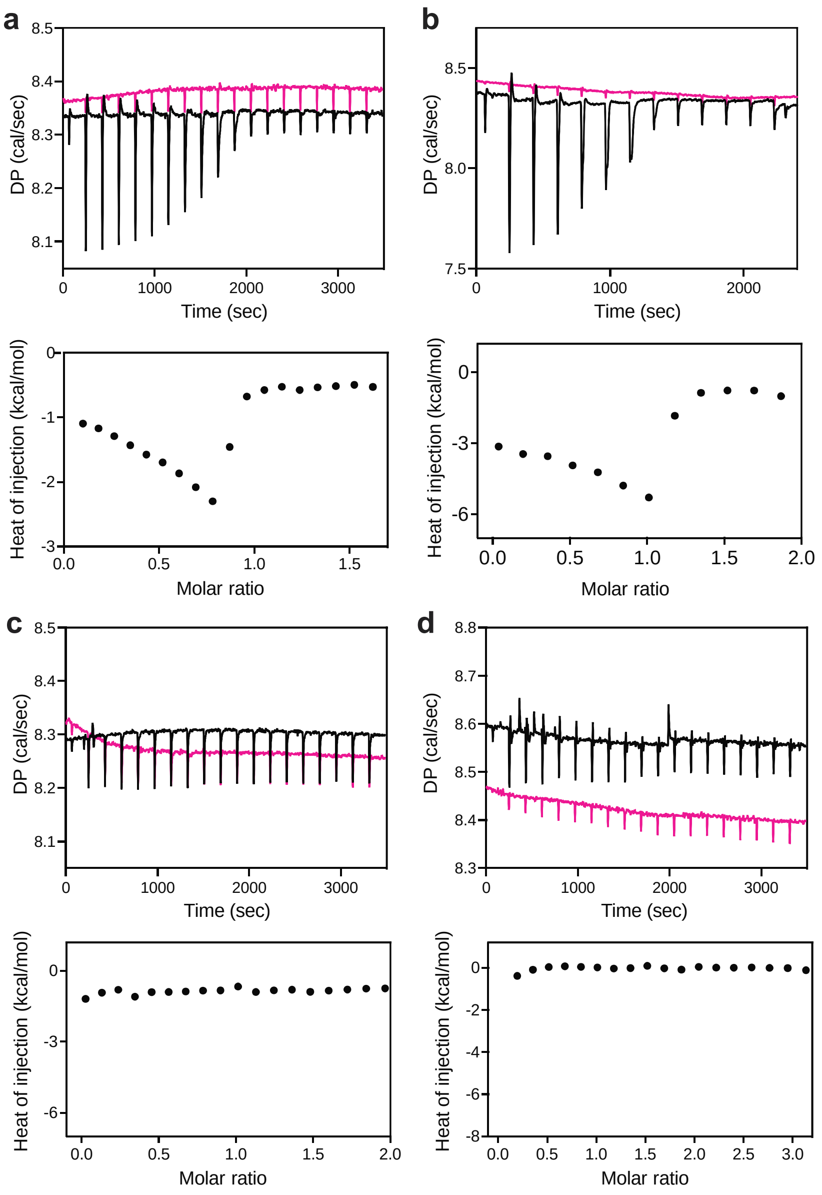


**Supplementary Figure 4 | ITC data used for SVD and elucidating ligand binding.** The raw thermograms and isotherms from two independent ITC experiments of rCbl (**a-b**) and single ITC experiments for two kissing-loop modified mutants M2 and M3 (**c-d**). Panel (**a**) corresponds to **Fig. 2f** and panels (**b-d**) correspond to **Fig. 4d**. Thermograms of ligand-to-RNA and ligand-to-buffer titrations are colored in black and magenta, respectively. Titrations with rCbl and M3 were performed twice. The titration with M2 was performed only once since the deletion of P13 is a more extreme approach to abolishing the KL interaction, whose effect is sufficiently demonstrated in M3 which has only disrupting mutations in L13. Source data are provided as a Source Data file.

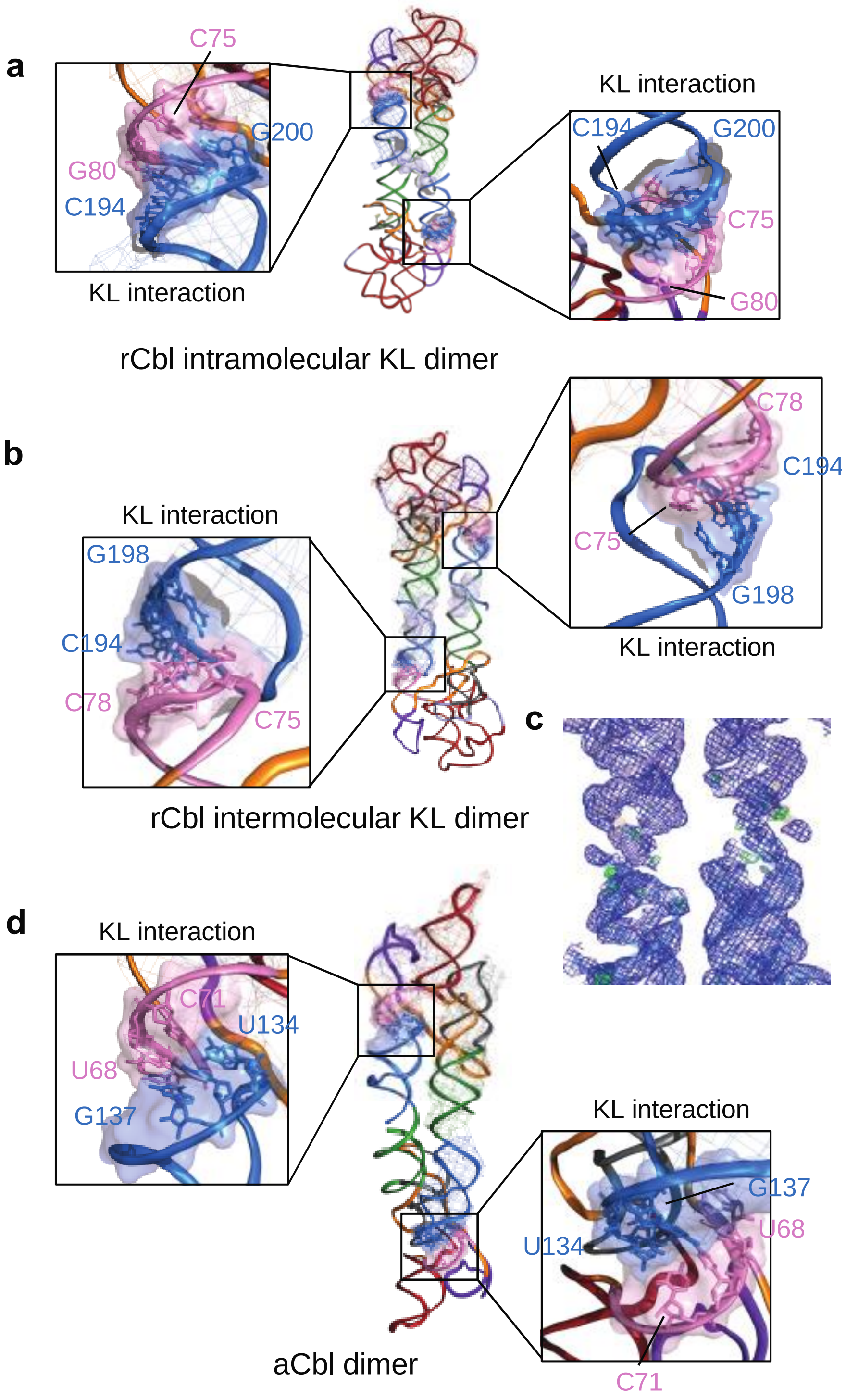


**Supplementary Figure 5 | Cobalamin riboswitch kissing-loop interactions.** Cartoon illustrations of the kissing-loop interactions between L5 (pink) and L13 (blue), as observed in the crystal structure of rCbl (PDB: 4GMA) interpreted as a dimer with (**a**) intramolecular kissing loops or (**b**) intermolecular kissing loops. (**c**) Electron density map for the dimer interface (blue: $2F_o$-$F_c$ ($1\sigma$); green: $F_o$-$F_c$ ($3\sigma$)), with no additional structure modelling or refinement, showing indications of an intermolecular KL dimer. RNA domains are colored as follows P1 (green), P2 (gray), P3 (orange), P4 (purple), P5 (pink), P6 extension (red), P13 (blue). (**d**) atypical cobalamin riboswitch (aCbl) dimer (PDB: 6VMY). The meshed region in panels a, b, and d, outlines a single monomer.

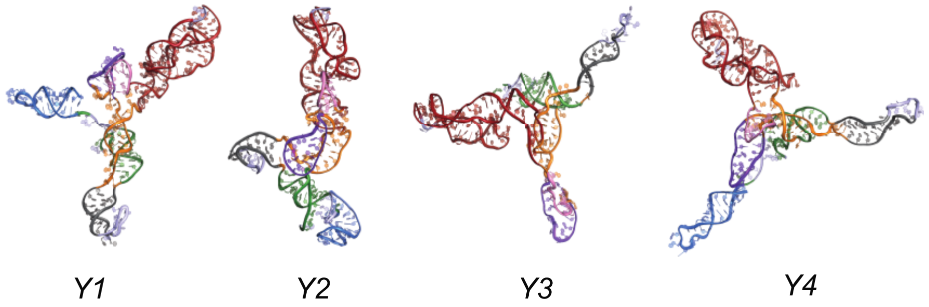


**Supplementary Figure 6 | Recapitulated structures of *Y* conformers.** Cartoon models of the recapitulated structures of *Y1* and *Y2* conformers (rCbl) and *Y3* and *Y4* (rCbl mutants M2 and M3, respectively). Domains P1, P2, P3, P4, P5, P6 extension, and P13 are colored in green, gray, orange, purple, pink, red, and blue, respectively.

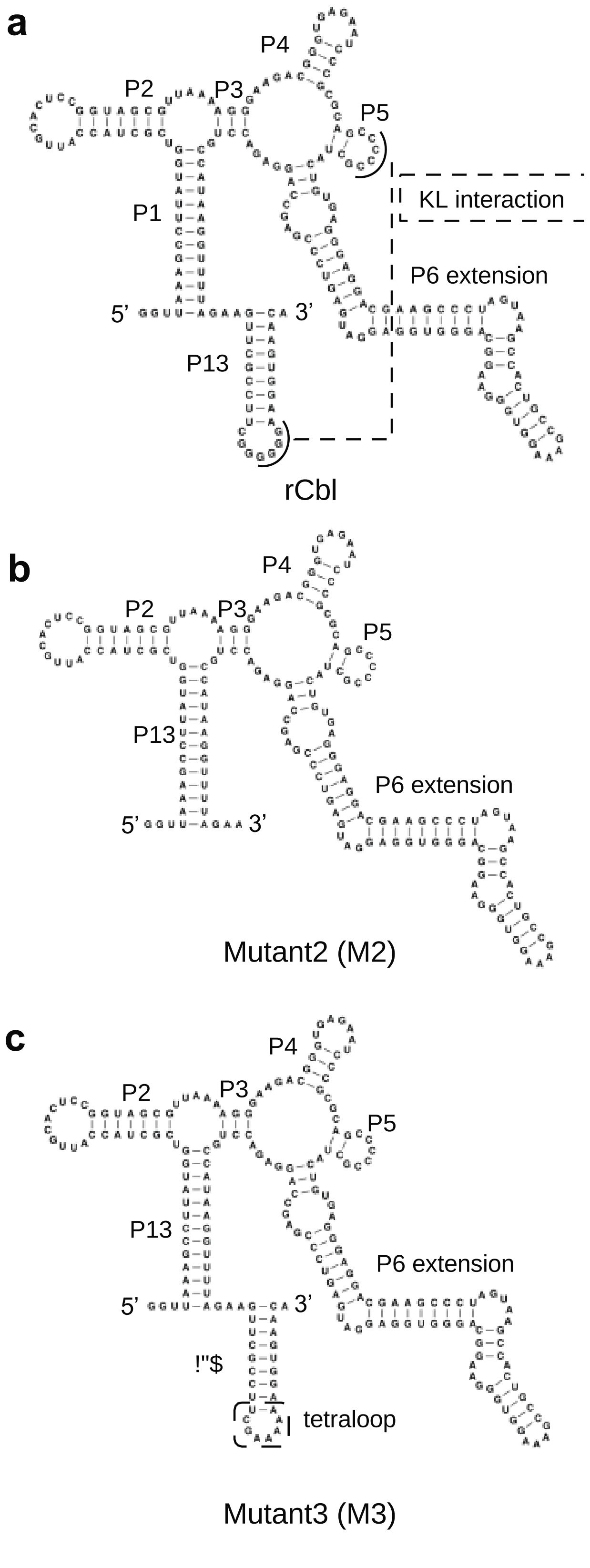
a
P4
P2
P3
P5
KL interaction
P1
P6 extension
5’
3’
P13
rCbl
b
P4
P2
P3
P5
P13
P6 extension
5’
3’
Mutant2 (M2)
c
P4
P2
P3
P5
P13
P6 extension
5’
3’
!"$
tetraloop
Mutant3 (M3)

**Supplementary Figure 7 | The secondary structures of rCbl and mutants**. The rCbl (**a**), and mutants M2 (**b**) and M3 (**c**) secondary structures. In M2, the P13 helix is deleted entirely. In M3, L13 (GGGGG) is replaced with a tetraloop (GAAA). Both mutants are designed to disrupt the L5-L13 KL interaction.

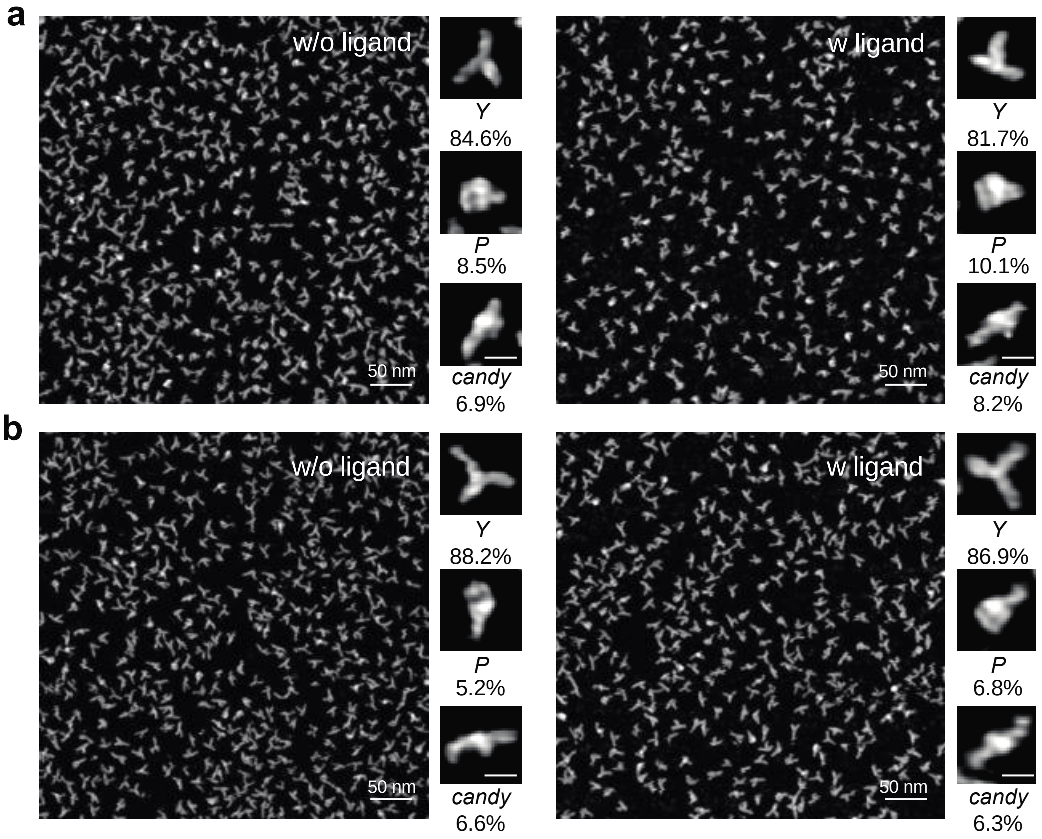


**Supplementary Figure 8 | AFM imaging of rCbl mutants that abrogate the KL interactions.** AFM images of M2 (**a**) and M3 (**b)** in the absence (left) or presence (right) of 1 mM AdoCbl. The same three types of conformations (*Y*, *P*, *candy*) with nearly identical populations were observed in all images. Insets show representative particles for each conformer type and their respective populations. The scale bars shown in the right bottom corner of cropped particles are 10 nm. The total number of images recorded for each sample were as follows: M2 w/o ligand (3), M2 with ligand (4), M3 w/o ligand (4), M3 with ligand (5).

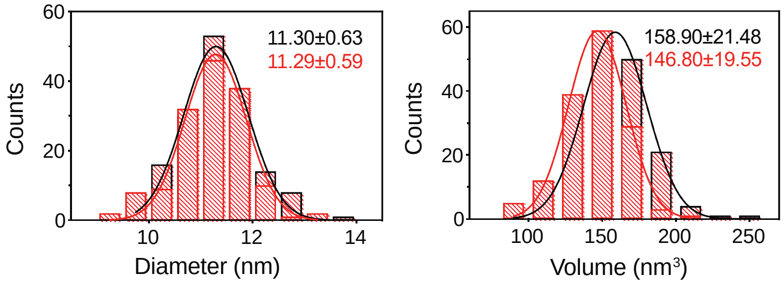


**Supplementary Figure 9 | Particle diameter and volume histograms.** Particle diameters (left) and particle volumes (right) for *Y* conformers of M2 (black, 160 particles) and M3 (red, 148 particles) determined using SPIP (Metrology). The Gaussian fits with mean values and standard deviations are shown. Source data are provided as a Source Data file.

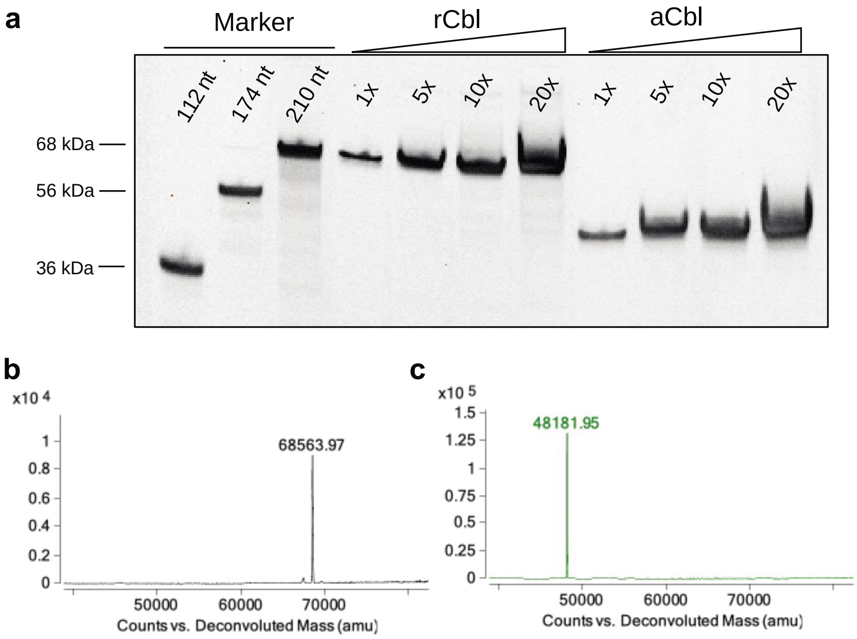


**Supplementary Figure 10 | Confirmation of size and purity of rCbl and aCbl.** (**a**) 6% denaturing PAGE gel showing single bands at the expected lengths. Serially diluted samples were loaded onto the gel with minimal 13.7 μg (1x) for rCbl and 12.2 μg (1x) for aCbl. The gel was stained by Sybr Gold. Source data are provided as a Source Data file. (**b-c**) Deconvoluted mass spectrometry data reflecting the theoretical molecular weights (rCbl: 68557.2 Da, aCbl: 48175.9 Da).

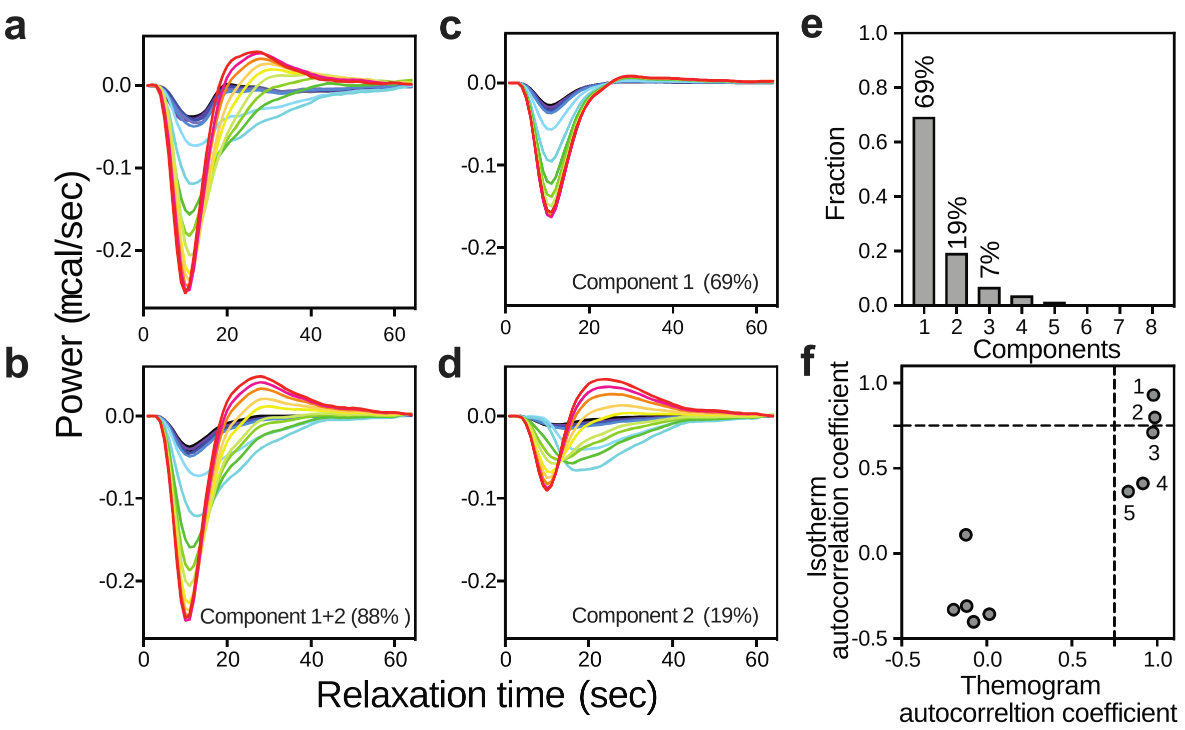


**Supplementary Figure 11 | SVD analysis of the ITC thermogram. (a)** The raw ITC thermogram shown in **Supplementary Fig. 4a** was deconvoluted and reconstructed using a minimum of two principal components determined by SVD. The heat compensation profiles were color-ramped from red to deep blue corresponding to each titration point. **(b)** The SVD-reconstructed thermogram corresponding to the two major components represents 88% of the total singular values. The SVD-deconvoluted thermograms for Components 1 and 2 are shown in panels **(c)** and **(d)**, respectively. **(e)** The percentage of singular values and **(f)** their related autocorrelation coefficients determined by SVD analysis. Source data are provided as a Source Data file.

# Supplementary Tables

**Supplementary Table 1**. Coarse-grained dynamic fitting statistics for rCbl topological structure calculations for each monomer and dimer class. Rg: radius of gyration; E(covalent): energy of bond length, angle and dihedral angle; E(stack): energy of base-stacking interaction; E(HB): energy of hydrogen bond; E(repul): energy of repulsive interaction; E(elect): energy of electric static interaction; E(AFM): pseudo potential energy of AFM; afmCC: cross-correlation between the AFM image and a structure.

| | Rg | E(total) | E(covalent) | E(contact) | E(stack) | E(HB) | E(repul) | E(elect) | E(AFM) | afmCC |
|---|---|---|---|---|---|---|---|---|---|---|
| ***compact*** | 30.79 | -3896.85 | 568.5 | -464.34 | -2589.01 | -1984.18 | 1.93 | 235.29 | 333.97 | 0.9942886 |
| ***candy1*** | 38.56 | -231.35 | 489.78 | -623.03 | -221.03 | -181.97 | 2.98 | 216.49 | 84.45 | 0.9924422 |
| ***candy2*** | 39.24 | -4085.23 | 494.14 | -368.7 | -2563.82 | -1935.54 | 2.51 | 183.32 | 101.87 | 0.9875678 |
| ***P*** | 32.02 | -3178.03 | 562.81 | -595.13 | -1994.8 | -1516.29 | 0.97 | 217.67 | 145.76 | 0.9869547 |
| ***FF*** | 57.40 | -17182.42 | 980.25 | -1903.39 | -4398.86 | -12583.76 | 6.1 | 526.9 | 194.57 | 0.982614 |
| ***OLO*** | 59.71 | -9200.1 | 1073.7 | -3102.86 | -3879.11 | -4385.09 | 12.66 | 479.47 | 600.14 | 0.9884903 |
| ***SS*** | 44.47 | -26044.27 | 1161 | -8601.29 | -5219.31 | -15916.64 | 58.5 | 585.19 | 1898.73 | 0.9494404 |
| ***PY*** | 56.94 | -16979.12 | 1061.31 | -5234.3 | -3856.45 | -9786.39 | 10.28 | 424.38 | 401.06 | 0.9907171 |
| ***Y1**** | 45.02 | -3614.53 | 611.7 | -240.09 | -2412.63 | -1908.31 | 1.6 | 179.64 | 152.56 | 0.9918077 |
| ***Y2**** | 44.59 | -5091.41 | 595.01 | -216.66 | -3100.2 | -2635.08 | 2.52 | 169.82 | 92.2 | 0.9973666 |
| ***Y3***** | 44.94 | -2750.53 | 483.53 | -191.51 | -1948.34 | -1975.28 | 1.66 | 131 | 747.47 | 0.930575 |
| ***Y4***** | 49.12 | -4377.74 | 545.91 | -265.68 | -1931.64 | -2998.29 | 3.08 | 157.79 | 110.12 | 0.9729926 |

* Conformers refer to *Y*-conformers *Y1* and *Y2* of rCbl.
** Conformers refer to those observed for rCbl mutants, M2 and M3, respectively.

**Supplementary Table 2**. Isothermal titration calorimetry (ITC)-derived thermodynamic parameters for the interaction of rCbl with AdoCbl after singular value decomposition (SVD) (**Fig. 2f**).

| | | $K_d$ (nM) | $N_{app}$ | ΔG (kcal/mol) | ΔH (kcal/mol) | -ΔST (kcal/mol) |
|---|---|---|---|---|---|---|
| **component 1** | 69% | 626.2±40.5 | 0.76* | -8.46 | -1.69±0.04 | -6.77 |
| | | 600.6±69.1 | 0.78 | -8.56 | -1.68±0.03 | -6.80 |
| **component 2** | 19% | 1347±266† | 0.65* | -7.85 | 4.03±0.24 | -12.03 |
| | | 1738±510† | 0.71 | -7.85 | 4.46±1.15 | -12.31 |
| | | 85.0±54.4† | 0.88* | -9.64 | -1.69±0.13 | -7.95 |
| | | 136.4±120† | 0.89 | -9.36 | -2.90±1.15 | -6.64 |

* Stoichiometric $N_{app}$ (component 1) and mean $N_{app}$ (component 2) value of 0.76 used for fitting based on the AFM fraction of conformers in the absence of ligand that were not *Y*-shaped (76%) (**Fig. 2e**).
† Standard error greater than 10% of the mean.

**Supplementary Table 3.** Validation of recapitulated structures using RNase P RNA core domain.

| noise (%) | RMSD (Å) best_str | std.dev (Å) | RMSD (Å) top_str | std.dev (Å) std.dev (Å) | CC | std.dev |
|---|---|---|---|---|---|---|
| 5 | 4.28 | 0.11 | 5.04 | 0.26 | 0.996918 | 0.000153 |
| 10 | 4.25 | 0.10 | 4.96 | 0.19 | 0.995953 | 0.000186 |
| 15 | 3.91 | 0.05 | 5.10 | 0.39 | 0.995738 | 0.000244 |
| 20 | 4.84 | 0.01 | 5.69 | 0.48 | 0.994756 | 0.000197 |
| 30 | 4.52 | 0.03 | 5.14 | 0.45 | 0.993116 | 0.000213 |
| 40 | 4.46 | 0.07 | 5.67 | 0.99 | 0.987446 | 0.000176 |
| 50 | 4.36 | 0.04 | 6.29 | 0.13 | 0.973870 | 0.000140 |
| 60 | 4.68 | 0.04 | 6.87 | 0.93 | 0.951720 | 0.000190 |
| 70 | 5.99 | 0.07 | 7.13 | 0.36 | 0.921000 | 0.000300 |
| 80 | 5.71 | 0.05 | 6.70 | 0.74 | 0.888260 | 0.000260 |
| | | | 7.39 | 0.27 | 0.887750 | 0.000280 |
| 90 | 8.45 | 0.06 | 10.32 | 0.53 | 0.847320 | 0.000470 |
| 100 | 9.71 | 0.03 | 14.34 | 0.27 | 0.809747 | 0.000650 |

Note: at the 80% noise level, two calculations using different weighting factors $\theta^{AFM}$ score equally.

**Supplementary Table 4**. Molecular weights estimated from experimental SAXS data.

| | $R_g$ Guinier (Å) | $R_g$ real space (Å) | $D_{max}$ (Å) | MW ($V_c$)* (kDa) | MW (fit)† (kDa) | Monomer MW (kDa) |
|---|---|---|---|---|---|---|
| **rCbl (no ligand)** | 58.9±2.4 | 61.1±1.1 | 207 | 86.7 | 99.8 | 68.5 |
| **rCbl (1mM ligand)** | 57.1±2.2 | 59.0±1.1 | 197 | 97.7 | 108.0 | 70.0 |

* MW ($V_c$): Mean particle weight derived from experimental SAXS data using method based on correlation volume for RNA at $Q_{max}$=0.3 $Å^{-1}$.

† The MWs (fit) are calculated using the dimer and monomer volume percentages from the SAXS fitting results, plus the theoretical MWs for monomer and dimer, as the equation shown below:

MW(fit) = dimer volume % x theoretical dimer MW + monomer volume % x theoretical monomer MW (**Fig. 2e**).

**Supplementary Table 5.** Volume and radius of gyration ($R_g$) values for various conformers.

| | *Y* | *candy* | *P* | *compact* | *FF* | *OLO* | *SS* | *P-Y* | *P-candy* |
|---|---|---|---|---|---|---|---|---|---|
| **Volume (nm$^3$)** | 98.3 | 99.8 | 98.4 | 102.1 | 216.5 | 209.4 | 195.5 | 213.5 | 196.7 |
| **$R_g$ (Å)** | 44.8 | 38.7 | 38.6 | 34.2 | 57.4 | 62.5 | 44.3 | 54.6 | 62.0 |

Note:
The volume values were assessed using 3V Web Server (http://3vee.molmovdb.org/)[4] by setting the probe radius to 5.0 Å. $R_g$ values were calculated using CRYSOL3[5]

**Supplementary Table 6.** SAXS data acquisition, sample details, data analysis and software used.

(a) Sample details

| | rCbl | rCbl+AdoCbl |
|---|---|---|
| Organism | *Thermoanearobacter tengcongensis* | *Thermoanearobacter tengcongensis* |
| Source (Catalogue No. or reference) | *in vitro* transcription | *in vitro* transcription |
| Description: sequence (including Uniprot ID + uncleaved tags), bound ligands/modifications, *etc.* | GGUUAAAGCCUUAUGG UCGCUACCAUUGCACUC CGGUAGCGUUAAAAGG GAAGACGGGUGAGAAU CCCGCGCAGCCCCCGCU ACUGUGAGGGAGGACG AAGCCCUAGUAAGCCAC UGCCGAAAGGUGGGAA GGCAGGGUGGAGGAUG AGUCCCGAGCCAGGAGA CCUGCCAUAAGGUUUUA GAAGUUCGCCUUCGGGG GGAAGGUGAACA | GGUUAAAGCCUUAUGG UCGCUACCAUUGCACUC CGGUAGCGUUAAAAGG GAAGACGGGUGAGAAU CCCGCGCAGCCCCCGCU ACUGUGAGGGAGGACG AAGCCCUAGUAAGCCAC UGCCGAAAGGUGGGAA GGCAGGGUGGAGGAUG AGUCCCGAGCCAGGAGA CCUGCCAUAAGGUUUUA GAAGUUCGCCUUCGGGG GGAAGGUGAACA |
| Extinction coefficient ε (wavelength and units) | 0.0382 $(\mu g/ml)^{-1}cm^{-1}$ (A260) | 0.0382 $(\mu g/ml)^{-1}cm^{-1}$ (A260) |
| Molecular mass *M* from chemical composition (Da) | 68478.2 | 70057.78 |
| Concentration (range/values) measured | 0.025~2 μM | 0.025~2 μM |
| Solvent composition and source | 50 mM MES, pH 6.0, 10 mM KCl, 1 mM $MgCl_2$ | 50 mM MES, pH 6.0, 10 mM KCl, 1 mM $MgCl_2$, 0.225~18 uM unbound AdoCbl |

(b) SAS data collection parameters

| | |
|---|---|
| Source, instrument and description or reference | 12ID-B beamline of the Advanced Photon Source (APS), Argonne National Laboratory |
| Wavelength (Å) | 0.932 |
| Beam geometry (size, sample-to-detector distance) | 1.9 m |
| q-measurement range ($Å^{-1}$) | 0.005-0.88 |
| Absolute scaling method | A relative scale was used |
| Basis for normalization to constant counts | to transmitted intensity by beam-stop counter |
| Method for monitoring radiation damage, X-ray dose where relevant | data frame-by-frame comparison |
| Exposure time, number of exposures | 1s per frame and 225 frames in total |
| Sample configuration including path length and flow rate where relevant | flow cell with 1.5mm path length and flow rate of 10 μL/s |
| Sample temperature (ºC) | 10 |

(c) Software employed for SAS data reduction, analysis and interpretation

| | |
|---|---|
| SAS data reduction | MATLAB codes developed by APS 12-ID-B, solvent subtraction using Argonne SAXS macros in Igor Pro |
| Calculation of ε from sequence | using online Oligo Calc: http://biotools.nubic.northwestern.edu/OligoCalc.html |
| Basic analyses | Guinier, P(r), PRIMUSqt from ATSAS 3.0.3 (Petoukhov et al., 2012) $V_c$:Scatter |
| Atomic structure modelling | Crysol 3.0 |

(d) Structural parameters

| Guinier Analysis | rCbl | rCbl+AdoCbl |
|---|---|---|
| I(0) (a.u.) | 0.037±0.001 | 0.039±0.001 |
| $R_g$ (Å) | 58.9±2.4 | 57.1±2.2 |
| $q_{min}$ (Å$^{-1}$)qmin | 0.005 | 0.005 |
| qRg max | 1.29 | 1.29 |
| Coefficient of correlation, $R^2$ | 1 | 1 |
| MW from $V_c$ (ratio to expected value) | 86.7kDa (1.27) | 97.7kDa (1.4) |
| Volume ($V_c$) | 843.9 | 893.9 |
| **P(r) analysis** | **rCbl** | **rCbl+AdoCbl** |
| I(0) (a.u.) | 0.037±0.001 | 0.038±0.001 |
| $R_g$ (Å) | 61.1±1.1 | 59.0±1.1 |
| $d_{max}$ (Å) | 207 | 197 |
| q-range (Å$^{-1}$) | 0.005~0.3 | 0.005~0.3 |
| MW from $V_c$ for RNA at Qmax=0.3 Å$^{-1}$ (ratio to expected value) | 84.3(1.23) | 92.4(1.32) |
| Volume ($V_c$) | 843.9 | 878.4 |

(e) Data and model deposition IDs[a]

| rCbl | rCbl+AdoCbl |
|---|---|
| SASDQG7 https://www.sasbdb.org/data/SASDQG7/oyqh9rskld | SASDQH7 https://www.sasbdb.org/data/SASDQH7/74furjoaj1 |

**Note:**

**a.** The SAXS data recorded using the samples containing the mixture of monomeric, dimeric and higher order oligomer species, each with various conformations could not be fit using a single structure but multiple structures with weighted volume fractions as described in this study. As a result, the fit data set could not be deposited using the standard format on the SASBDB site. Nevertheless, we made effort and deposited as much information as possible at the site as the following:

1. In order to make the fitting data plotted correctly by SASbdb, the fitting data has to be modified in a format that is supported by SASbdb. The experimental data were interpolated to have the same q values as that from the fitting model. The fitting data were formatted in 4 columns (Q, Iexpt, Error, Ifit) and a specific format of the header is added. Only Chi2 in the header is relevant.

2. The SASBDB allows uploading only up to three fitting curves and their corresponding models. As a result, we could only deposit the synthesized curves and two fitting curves from two individual conformation models together with all other models for the apo and holo states. Thus, in order to have the complete sets of data in one place, we made all curves back-calculated from all conformers available at our website: https://home.ccr.cancer.gov/csb/pnai/data/InfoForRNAHeterogeneityStudy/SAXS_plots/

## Supplementary References


1 Shlyakhtenko, L. S. *et al.* Silatrane-based surface chemistry for immobilization of DNA, protein-DNA complexes and other biological materials. *Ultramicroscopy* **97**, 279-287, doi:10.1016/S0304-3991(03)00053-6 (2003).

2 Lyubchenko, Y. L., Shlyakhtenko, L. S. & Gall, A. A. Atomic force microscopy imaging and probing of DNA, proteins, and protein DNA complexes: silatrane surface chemistry. *Methods Mol Biol* **543**, 337-351, doi:10.1007/978-1-60327-015-1_21 (2009).

3 Stumme-Diers, M. P., Stormberg, T., Sun, Z. & Lyubchenko, Y. L. Probing The Structure And Dynamics Of Nucleosomes Using Atomic Force Microscopy Imaging. *J Vis Exp*, doi:10.3791/58820 (2019).

4 Voss, N. R. & Gerstein, M. 3V: cavity, channel and cleft volume calculator and extractor. *Nucleic Acids Res* **38**, W555-562, doi:10.1093/nar/gkq395 (2010).

5 Franke, D. *et al.* ATSAS 2.8: a comprehensive data analysis suite for small-angle scattering from macromolecular solutions. *J Appl Crystallogr* **50**, 1212-1225, doi:10.1107/S1600576717007786 (2017).
