# Supplementary material for: Visualizing RNA conformational and architectural heterogeneity in solution
Source: Nat Commun. 2023 Feb 9;14:714. doi: 10.1038/s41467-023-36184-x (PMC9911696; doi:10.1038/s41467-023-36184-x)
Supplement: Supplementary file 3 — Reporting Summary [file 41467_2023_36184_MOESM3_ESM.pdf]

nature portfolio

Corresponding author(s): Yun-Xing Wang

Last updated by author(s): Jan 5, 2023

Reporting Summary

Nature Portfolio wishes to improve the reproducibility of the work that we publish. This form provides structure for consistency and transparency in reporting. For further information on Nature Portfolio policies, see our Editorial Policies and the Editorial Policy Checklist.

Statistics

For all statistical analyses, confirm that the following items are present in the figure legend, table legend, main text, or Methods section.

n/a	Confirmed	
☐	☒	The exact sample size (*n*) for each experimental group/condition, given as a discrete number and unit of measurement
☒	☐	A statement on whether measurements were taken from distinct samples or whether the same sample was measured repeatedly
☐	☒	The statistical test(s) used AND whether they are one- or two-sided<br>*Only common tests should be described solely by name; describe more complex techniques in the Methods section.*
☒	☐	A description of all covariates tested
☒	☐	A description of any assumptions or corrections, such as tests of normality and adjustment for multiple comparisons
☐	☒	A full description of the statistical parameters including central tendency (e.g. means) or other basic estimates (e.g. regression coefficient) AND variation (e.g. standard deviation) or associated estimates of uncertainty (e.g. confidence intervals)
☒	☐	For null hypothesis testing, the test statistic (e.g. *F*, *t*, *r*) with confidence intervals, effect sizes, degrees of freedom and *P* value noted<br>*Give P values as exact values whenever suitable.*
☒	☐	For Bayesian analysis, information on the choice of priors and Markov chain Monte Carlo settings
☒	☐	For hierarchical and complex designs, identification of the appropriate level for tests and full reporting of outcomes
☒	☐	Estimates of effect sizes (e.g. Cohen's *d*, Pearson's *r*), indicating how they were calculated

Our web collection on statistics for biologists contains articles on many of the points above.

Software and code

Policy information about availability of computer code

Data collection	No custom software was used for data collection.
Data analysis	Custom MATLAB scripts were used (MATLAB Statistical Tool Box Version 2021a) as well as GraphPad Prism (v. 9.4.1) were used for SVD analysis and fitting. Model and computation data and information are available at https://home.ccr.cancer.gov/csb/pnai/data/InfoForRNAHeterogeneityStudy/

For manuscripts utilizing custom algorithms or software that are central to the research but not yet described in published literature, software must be made available to editors and reviewers. We strongly encourage code deposition in a community repository (e.g. GitHub). See the Nature Portfolio guidelines for submitting code & software for further information.

Data

Policy information about availability of data

All manuscripts must include a data availability statement. This statement should provide the following information, where applicable:

- Accession codes, unique identifiers, or web links for publicly available datasets
- A description of any restrictions on data availability
- For clinical datasets or third party data, please ensure that the statement adheres to our policy

Source data are provided with this paper. The SAXS data generated in this study have been deposited in the SASBDB under accession codes SASDQG7 (https://www.sasbdb.org/data/SASDQG7/oyqh9rskld) and SASDQG7 (https://www.sasbdb.org/data/SASDQH7/74furjoaj1) for rCbl in the absence and presence ligand,

respectively. All back-calculated SAXS curves based on observed conformers together with the synthesized SAXS and experimental SAXS curves are available at https://home.ccr.cancer.gov/csb/pnai/data/InfoForRNAHeterogeneityStudy/SAXS_plots/. All structural model coordinates and computation files for rCbl are available at https://home.ccr.cancer.gov/csb/pnai/data/InfoForRNAHeterogeneityStudy/. PDB coordinates for rCbl, aCbl, and RNase P used for the analyses in this study are 4GMA (https://www.rcsb.org/structure/4GMA), 6VMY (https://www.rcsb.org/structure/6VMY), and 3DHS (https://www.rcsb.org/structure/3DHS), respectively.

Human research participants

Policy information about studies involving human research participants and Sex and Gender in Research.

Reporting on sex and gender	N/A
Population characteristics	N/A
Recruitment	N/A
Ethics oversight	N/A

Note that full information on the approval of the study protocol must also be provided in the manuscript.

Field-specific reporting

Please select the one below that is the best fit for your research. If you are not sure, read the appropriate sections before making your selection.

☒ Life sciences ☐ Behavioural & social sciences ☐ Ecological, evolutionary & environmental sciences

For a reference copy of the document with all sections, see nature.com/documents/nr-reporting-summary-flat.pdf

Life sciences study design

All studies must disclose on these points even when the disclosure is negative.

Sample size	A sample size of 225 measurements were used for SAXS data analysis.
Data exclusions	Two of 225 measurements in each data set were omitted as outliers.
Replication	SAXS data were collected as a series of measurements on each sample.
Randomization	No statistical analysis in this study required the use of randomization for collecting small-angle x-ray scattering data.
Blinding	No statistical analysis in this study required the use of blinding for collecting small-angle x-ray scattering data.

Reporting for specific materials, systems and methods

We require information from authors about some types of materials, experimental systems and methods used in many studies. Here, indicate whether each material, system or method listed is relevant to your study. If you are not sure if a list item applies to your research, read the appropriate section before selecting a response.

Materials & experimental systems

n/a	Involved in the study	
☒	☐	Antibodies
☒	☐	Eukaryotic cell lines
☒	☐	Palaeontology and archaeology
☒	☐	Animals and other organisms
☒	☐	Clinical data
☒	☐	Dual use research of concern

Methods

n/a	Involved in the study	
☒	☐	ChIP-seq
☒	☐	Flow cytometry
☒	☐	MRI-based neuroimaging
